# Correlation of anthropometric indicators for identifying insulin sensitivity and resistance

**DOI:** 10.1590/S1516-31802011000100006

**Published:** 2011-01-06

**Authors:** Lívia Nascimento Matos, Guilherme de Vieira Giorelli, Cristiane Bitencourt Dias

**Affiliations:** I MD. Postgraduate student, Department of Internal Medicine, Institute for Medical Treatment, Hospital do Servidor Público Estadual de São Paulo – Francisco Morato de Oliveira, São Paulo, Brazil.; II MD, PhD. Attending Physician, Department of Internal Medicine, Institute for Medical Treatment, Hospital do Servidor Público Estadual de São Paulo – Francisco Morato de Oliveira, São Paulo, Brazil.

**Keywords:** Insulin resistance, Prediabetic state, Hyperglycemia, Body mass index, Waist circumference, Anthropometry, Resistência à insulina, Estado pré-diabético, Hiperglicemia, Índice de massa corporal, Circunferência da cintura, Antropometria

## Abstract

**CONTEXT AND OBJECTIVES:**

Early detection of reduced insulin sensitivity (IS) and insulin resistance (IR) is desirable. The aim here was to evaluate correlations of anthropometric indicators for identifying IR or IS and determine the cutoff points of the most effective indicators.

**DESIGN AND SETTING:**

Cross-sectional study in the city of São Paulo.

**METHODS:**

Sixty-one individuals with normal fasting plasma glucose (NFPG) and 43 overweight women were analyzed. Body mass index (BMI), waist circumference (WC), waist-to-hip ratio, waist-to-height ratio (WHtR), conicity index and the HOMA-IS and HOMA-IR indices were determined. The correlations between the anthropometric indices and IS and IR were determined. ROC analysis was used to determine the areas under the curve (AUC) and cutoff points.

**RESULTS:**

Among the NFPG individuals, BMI (r = -0.50; P = 0.002) and WHtR (r = -0.45; P = 0.007) showed correlations with HOMA-IS (homeostasis model assessment of insulin sensitivity). The ROC curve demonstrated statistical significance for BMI (AUC = 0.769; P = 0.005), WHtR (AUC = 0.764; P = 0.01) and WC (AUC = 0.702; P = 0.04), and the best cutoff points were 33.3 kg/m^2^, 0.67 and 100 cm, respectively. Among the overweight women, the best correlation with HOMA-IR was demonstrated by WHtR (r = 0.37; P = 0.01), and the best cutoff point was 0.70 (AUC = 0.61; P = 0.25).

**CONCLUSION:**

The most promising indicators for showing IS among the NFPG individuals were BMI, WHtR and WC. Among the overweight women, WHtR demonstrated greater correlation with IR.

## RESUMO

**CONTEXTO E OBJETIVOS:**

A detecção precoce da redução na sensibilidade à insulina (SI) e resistência insulínica (RI) é desejável. O objetivo foi avaliar a correlação dos indicadores antropométricos em identificar a SI e RI, determinando os pontos de corte dos mais eficazes.

**TIPO DE ESTUDO E LOCAL:**

Estudo transversal na cidade de São Paulo.

**MÉTODOS:**

Analisou-se 61 indivíduos com glicemia de jejum normal (GJN) e 43 mulheres com sobrepeso. Determinou-se: índice de massa corporal (IMC), circunferência abdominal (CA), relação cintura quadril, relação cintura estatura (RCE), índice de conicidade e os índices HOMA-IS e HOMA-IR. As correlações entre os indicadores antropométricos e SI e RI foram determinadas. Análise ROC foi empregada com determinação das áreas abaixo da curva (AUC) e pontos de corte.

**RESULTADOS:**

No grupo de indivíduos com GJN, demonstraram correlação com o HOMA-IS (*homeostasis model assessment of insulin sensitivity*), o IMC (r = -0,50; P = 0,002) e RCE (= -0,45; P = 0,007). A curva ROC demonstrou significância estatística para IMC (AUC = 0,769; P = 0,005), RCE (AUC = 0,764; P = 0,01) e CA (AUC = 0,702; P = 0,04); os melhores pontos de corte foram 33,3 kg/m^2^, 0,67 e 100 cm, respectivamente. Entre mulheres com sobrepeso, as melhores correlações com o HOMA-IR foram demonstradas pela RCE (r = 0,37; P = 0,01), e o melhor ponto de corte foi 0,70 (AUC = 0,61; P = 0,25).

**CONCLUSÃO:**

Os indicadores mais promissores para indicar SI em indivíduos com GJN foram IMC, RCE e CA. Entre mulheres com sobrepeso, RCE demonstrou maior correlação com a RI.

## INTRODUCTION

Over recent decades, it has come to be considered that there is a worldwide pandemic of diabetes mellitus (DM). Data from the World Health Organization (WHO) indicate that the prevalence of DM is 2.8% among the worldwide population over 20 years of age.^1^ Predia-betes, characterized by abnormal fasting plasma glucose, glucose intolerance, or both, is often asymptomatic and the time that elapses between the early stages of these conditions and the diagnosing of DM ranges from four to seven years.^2^ Over this period, the complications relating to inadequate glucose metabolism progress and tissue damage becomes established before DM is diagnosed.^3-6^ Within this context, early detection of alterations in glucose metabolism is desirable, such that prophylactic interventions can be implemented.^7-9^

A prospective study demonstrated that reduced insulin sensitivity (IS), evaluated through the homeostasis model assessment of insulin sensitivity (HOMA-IS) index,^10^ was present five years before the appearance of abnormal fasting plasma glucose, glucose intolerance, or both, in previously normal individuals from the point of view of glucose metabolism. Moreover, during the transition from normal to abnormal metabolism, IS presented an additional decrease.^11^ Another index, called the homeostasis model assessment of insulin resistance (HOMA-IR), provides an indirect assessment of glucose metabolism, through evaluating endogenous insulin and plasma glucose homeostasis, as well as fasting plasma glucose.^12,13^

Obesity is a condition that involves a risk of such metabolic alterations.^14,15^ Therefore, anthropometric indicators among obese individuals are associated with a greater possibility of developing DM and metabolic syndrome. The indicators include body mass index (BMI),^16^ waist circumference (WC),^17^ waist-to-hip ratio (WHR),^18^ waist-to-height ratio (WHtR)^19^ and the conicity index (CI).^20^ However, such associations have been described both in normal healthy populations and in nutritionally heterogeneous populations.

## OBJECTIVE

The objective of the present study was to evaluate the correlation of anthropometric indicators for identifying abnormalities of glucose metabolism in a group of non-diabetic females who were overweight or presented abdominal and generalized obesity (evaluated through BMI and WC) and among individuals who were at risk of developing DM, but with normal fasting plasma glucose.

## MATERIALS AND METHODS

### Subjects and data collection

This was a cross-sectional study with a convenience sample, with analysis on prospectively collected data from individuals treated at the outpatient medical clinics of the Hospital do Servidor Público Estadual de São Paulo “Francisco Morato de Oliveira” between January and December 2009.

The study was approved by the research ethics committee of this hospital and the study subjects gave written informed consent (procedural number 0010.338.000-08).

The study included individuals with fasting plasma glucose ≤ 99 mg/dl and at least one of the following conditions that constitute a risk of developing DM: hypertension; BMI ≥ 25 kg/m^2^; high WC; first-degree kinship with diabetics; mothers of large-for-gestational age new-borns or who presented gestational DM; fasting serum high-density lipoprotein cholesterol (HDL) levels < 35 mg/dl and triglycerides > 250 mg/dl.^17^ Non-diabetic females with BMI ≥ 25 kg/m^2^ and WC ≥ 80 cm were also assessed separately. The exclusion criteria were a prior diagnosis of DM and use of oral hypoglycemic agents or insulin.

The study sample comprised 61 individuals with normal fasting plasma glucose levels and, in parallel, 43 females with BMI ≥ 25 kg/m^2^ and WC ≥ 80 cm. Most of these 43 women were undergoing outpatient follow-up treatment for hypertension and/or dyslipidemia.

### Measurements

We evaluated weight, height, WC, hip circumference, blood pressure (BP), presence of hypertension and dyslipidemia, along with the use of hypolipidemic drugs and hypotensors among the individuals included. BMI, WHR, WHtR and CI were calculated. The formulas used to calculate the indices studied are shown in **Table 1**.

**Table 1.** Formulas used in calculating the variables analyzed

Variable	Formula	References
Body mass index	weight (kg)/height (m)^2^	12
Waist-to-hip ratio	WC (cm)/Q (cm)	14
Waist-to-height ratio	WC (cm)/height (cm)	15
Conicity index	WC (m)/0.109 x √[weight (kg)/height (m)]	16
HOMA-IS	1/[insulin (mU/l) x glucose (mmol/l)/22.5]	10
HOMA-IR	insulin (mU/l) x glucose (mmol/l)/22.5	21

WC = waist circumference; Q = hip circumference; HOMA-IS = homeostasis model assessment of insulin sensitivity, HOMA-IR = homeostasis model assessment of insulin resistance.

All the data were evaluated by physicians with training on measurements of weight and height using standard techniques.^18^ WC was evaluated with the patient standing, at the end of exhalation, at the midpoint between the lower costal border and top of the iliac crest, using an inelastic tape horizontally.^19^ Hip circumference was measured at the level of the greater trochanter,^20,21^ in order to calculate WHR.^19,20,22^ BP was measured in accordance with the Seventh Report of the Joint National Committee on Prevention, Detection, Evaluation and Treatment of High Blood Pressure.^20^ Hypertension was defined as BP levels ≥ 140 x 90 mmHg on two different occasions, or situations of hypotensor use, regardless of BP levels.^20^ Diagnoses of dyslipidemia were evaluated in accordance with the laboratory criteria established in the Third Report of the National Cholesterol Education Program (NCEP) Expert Panel on Detection, Evaluation and Treatment of High Blood Cholesterol in Adults,^19^ or were defined as situations of hypolipidemic drug use, regardless of serum lipoprotein cholesterol levels and triglyceride levels.^19^ The study subjects underwent determinations of serum glucose levels and insulin levels after an 8 to 12-hour nocturnal fast. Plasma glucose was determined using the enzymatic method and insulin was determined using the immunometric method in a two-sided solid-phase chemiluminescence assay (Immulite 2000, Siemens). The HOMA-IS and HOMA-IR indices were determined through the formula shown in Table 1. Insulin sensitivity were considered to be preserved when HOMA-IS ≤ 0.37 and insulin resistance was considered to be present when HOMA-IR > 2.7, in accordance with a study on prevalence carried out among a Brazilian population.^23^

### Statistical analysis

Statistical analyses were performed using the MedCalc program, version 11.2. The level of statistical significance was set at P < 0.05. In order to evaluate the correlations of anthropometric data with HOMA-IS and HOMA-IR among the continuous variables, Pearson's coefficient was used on continuous variables with normal distribution and Spearman's coefficient was used on continuous variables that did not follow normal distribution. Fisher's exact test was used for categorical variables.

ROC (receiver operating characteristic) curves were constructed and the areas under the curve (AUC) were calculated, with a 95% confidence interval (CI).^16^ The Z test was used for comparisons of AUCs. Sensitivity (Sn) and specificity (Sp) values relating to detection of lower IS or higher IR were calculated for each cutoff point present in the sample. The cutoff value that presented the highest sum of Sn and Sp was chosen since it optimized the ratio between these two parameters.^24^

## RESULTS

The mean age of the 61 individuals with normal fasting plasma glucose was 59.7 ± 14.3 years, and 16 of them were males. The general characteristics of the sample studied are shown in **Table 2**, and it is noteworthy that the great majority reported hypertension and dyslipidemia, with mean systolic blood pressure of 132.5 ± 22.2 mmHg and diastolic arterial pressure of 80.9 ± 11.8 mmHg. Reduced insulin sensitivity was found in 25.6% of the patients, and the mean HOMA-IS for all patients was 1.0 ± 0.7, a value that was well above the level that is considered appropriate (≤ 0.37).

**Table 2.** Characterization of the individuals with normal fasting plasma glucose evaluated according to clinical, anthropometric and laboratory data

Categorical variables	%	n
Male gender	26.2%	16
Hypertension	62.2%	38
Dyslipidemia	67.2%	41

Continuous variables	Mean ± SD	Median (min-max)
Age (years)	59.7 ± 14.3	60 (18-83)
Weight (kg)	73.4 ± 13.3	71.2 (45.5-114.0)
Height (m)	1.5 ± 0.1	1.5 (1.42-1.96)
Body mass index (kg/m^2^)	29.5 ± 5.6	28.9 (19.43-43.44)
Systolic blood pressure (mmHg)	132.5 ± 22.2	130 (88-186)
Diastolic blood pressure (mmHg)	80.9 ± 11.8	80 (58-118)
Waist circumference (cm)	97.7 ± 13.8	98 (60-130)
Hip circumference (cm)	103.7 ± 15.8	103.5 (60-136)
Waist-to-hip ratio	0.9 ± 0.1	0.9 (0.7-1.7)
Waist-to-height ratio	0.6 ± 0.1	0.6 (0.3-0.8)
Conicity index	1.3 ± 0.1	1.3 (0.9-1.5)
Fasting plasma glucose (mg/dl)	91.7 ± 5.6	93 (77-99)
Fasting insulinemia (mUI/ml)	12.2 ± 19.4	5.0 (2-100)
HOMA-IS	1.0 ± 0.7	0.8 (0.04-2.44)

SD = standard deviation; min = minimum value; max = maximum value; HOMA-IS = homeostasis model assessment of insulin sensitivity.

**Figure 1.**
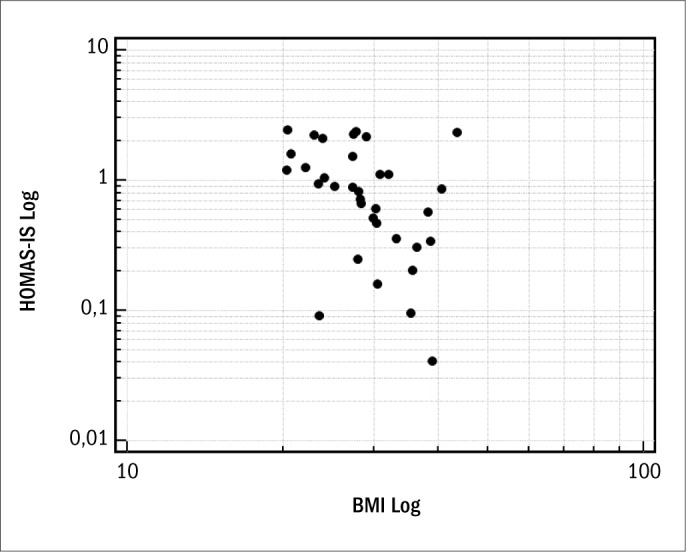
Spearman's correlation coefficient, after logarithmic transformation, between HOMA-IS (homeostasis model assessment of insulin sensitivity) and BMI (body mass index).

**Figure 2.**
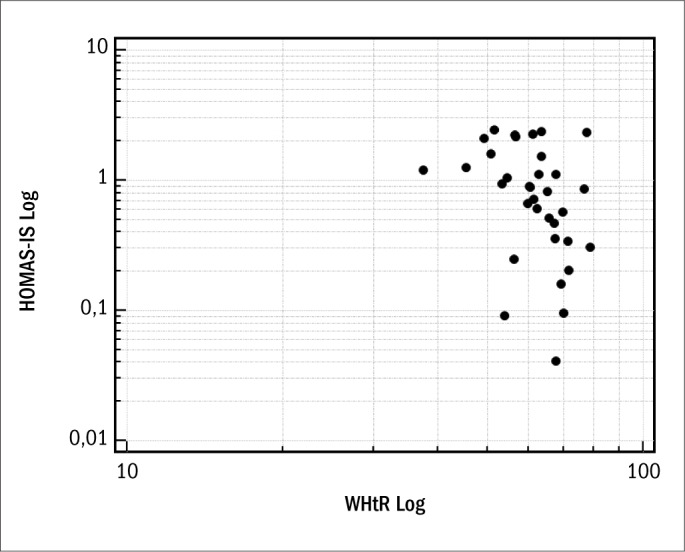
Spearman's correlation coefficient, after logarithmic transformation, between HOMA-IS (homeostasis model assessment of insulin sensitivity) and WHtR (waist-to-height ratio).

**Figure 3.**
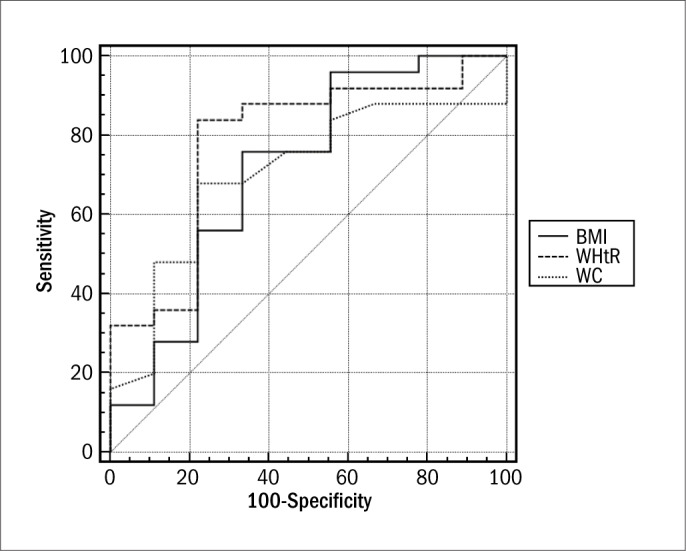
ROC (receiver operating characteristic) curve for the anthropometric indicators evaluated for assessing insulin sensitivity.

In parallel, we evaluated 43 non-diabetic females, of mean age 57.2 ± 12.9 years, who were either overweight or presented abdominal and generalized obesity. The prevalence of insulin resistance in the sample studied was 39.53%, with mean HOMA-IR of 3.8 ± 4.7, which were also abovenormal values (≤ 2.7). The general characteristics of the population studied are shown in **Table 3**. Regarding nutritional status, according to BMI data, 44.19% were overweight and 55.81% were obese. The correlation with HOMA-IS in the group of patients with normal fasting plasma glucose was demonstrated using BMI (r = -0.50; 95% CI: -0.72 to -0.19; P = 0.002) (**Figure 1**) and WHtR (r = -0.45; 95% CI: -0.684 to -0.132; P = 0.007) (**Figure 2**). A ROC curve was constructed for the anthropometric indicators evaluated and HOMA-IS was calculated in order to assess IS (**Figure 3**). Data on AUC, standard error (SE), 95% CI, cutoff points and the respective Sn and Sp demonstrated statistical significance in relation to BMI (AUC = 0.769; P = 0.005), WHtR (AUC = 0.764; P = 0.01) and WC (AUC = 0.702; P = 0.04), and the best cutoff points found were 33.3 kg/m^2^, 0.67 and 100 cm, respectively (**Table 4**).

**Table 3.** General characteristics of the group of obese non-diabetic females

Variables	Population studied (n = 43)
Age (years)	57.2 ± 13.0
HOMA-IR	3.8 ± 4.7
Body mass index (kg/m^2^)	32.5 ± 5.7
Waist circumference (cm)	103.6 ± 10.1
Waist-to-hip ratio	0.93 ± 0.06
Waist-to-height ratio	0.66 ± 0.06
Conicity index	1.33 ± 0.05

HOMA-IR = homeostasis model assessment of insulin resistance.

**Table 4.** Efficacy of the anthropometric indicators evaluated and fasting plasma glucose in assessing insulin sensitivity

Variables	Area ± SE (95% CI)	Cutoff point	Sensitivity (95% CI)	Specificity (95% CI)	Sn + Sp	P value
Body mass index	0.769 ± 0.096 (0.593 to 0.896)	30.33	80.00 (59.3 to 93.2)	77.78 (40.0 to 97.2)	157.78	0.005
Waist-to-height ratio	0.764 ± 0.107 (0.588 to 0.892)	0.67	84.00 (63.9 to 95.5)	77.78 (40.0 to 97.2)	161.78	0.01
Waist circumference	0.702 ± 0.101 (0.521 to 0.846)	100	68.00 (46.5 to 85.1)	77.78 (40.0 to 97.2)	145.78	0.04
Waist-to-hip ratio	0.584 ± 0.116 (0.403 to 0.750)	0.89	68.00 (46.5 to 86.1)	55.56 (21.2 to 86.3)	123.56	> 0.05
Conicity index	0.540 ± 0.125 (0.361 to 0.712)	1.355	56.00 (34.9 to 75.6)	77.78 (40.0 to 97.2)	133.78	> 0.05
Fasting plasma glucose	0.513 ± 0.122 (0.336 to 0.688)	89	80.00 (59.3 to 93.2)	44.44 (13.7 to 78.8)	124.44	> 0.05

SE = standard error; CI = confidence interval; Sn = sensitivity; Sp = specificity.

**Figure 4.**
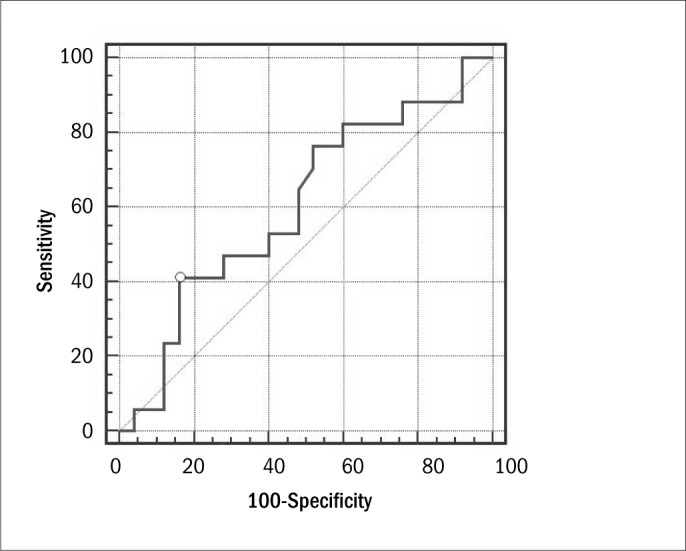
ROC (receiver operating characteristic) curve for waist-to-height ratio for assessing insulin resistance through the HOMA-IR (homeostasis model assessment of insulin resistance) index.

In the group of obese females, the most statistically significant correlation with the HOMA-IR index was demonstrated by the waist-to-height ratio (WHtR) (r = 0.37; P = 0.01; 95% CI: -0.6058 to -0.0822). The remaining anthropometric indicators of obesity and body composition that were evaluated did not demonstrate any significant correlations with the HOMA-IR index (P > 0.05). A ROC curve was constructed for WHtR, in order to assess IR, through HOMA-IR (**Figure 4**). In assessing the cutoff point with the greatest accuracy, WHtR reached the greatest sum between Sn and Sp values for the cutoff point 0.70 (AUC = 0.61 ± 0.09; P = 0.25) (**Table 5**).

**Table 5.** Efficacy of waist-to-height ratio for evaluating insulin resistance in the group of obese females

Variable	Area ± SE (95% CI)	Cutoff point	Sensitivity (95% CI)	Specificity (95% CI)	Sn + Sp	P
Waist-to-height ratio	0.61 ± 0.09 (0.44-0.75)	0.7	41.18 (18.4-67.1)	84.00 (63.9-95.5)	125.18	0.25

SE = standard error; CI = confidence interval; Sn = sensitivity; Sp = specificity.

## DISCUSSION

In the present study, BMI and WHtR demonstrated relevant negative correlations with HOMA-IS in individuals with normal fasting plasma glucose but presenting conditions that indicated that they were at risk of developing DM. The most promising anthropometric indicators for assessing IS were BMI, WHtR and WC. BMI and WC are widely used in clinical practice. However, WHtR still has not been incorporated into routine anthropometric assessment. Other research groups have already reported associations between WHtR and conditions such as left ventricular hypertrophy,^25^ hypertension,^26-28^ DM^27^ and insulin resistance in males classified as normal.^29^ Another important finding from the present study was the correlation between IR, evaluated through the HOMA-IR index, and WHtR among over-weight females.

Several studies have associated abdominal obesity with metabolic alterations and high cardiovascular risk, regardless of generalized obesity indicators.^30-32^ Imaging techniques such as nuclear magnetic resonance and computed tomography make it possible to observe different adipose tissue deposits at waist level. Among these are visceral and subcutaneous adiposity: the first of these is highly correlated with IS reduction and increased IR.^30-35^ In turn, WC has demonstrated a strong correlation with visceral adiposity and was therefore suggested by Lean et al. to be a cost-effective tool for such assessments.^36^

However, Hsieh and Yoshinaga demonstrated that individuals with similar WC values and lower height presented a worse metabolic and cardiovascular profile, demonstrated by greater hyperglycemia prevalence, hepatic steatosis and hypertension, compared with individuals with greater height, even after adjustment for age, smoking and lipid profile. This suggested that WHtR would be a more accurate tool in screening for the metabolic consequences of visceral deposits of adipose tissue.^37^ IS reduction and increased IR are subclinical conditions that have been considered to be precursor alterations of pre-diabetic status,^11^ which justifies active surveillance to diagnose such conditions. However, the laboratory analyses involved in this are expensive.

Therefore, every effort should be made towards determining cost-effective and easily interpreted criteria to identify such conditions. To this end, further studies should be encouraged in different populations, with the aim of validating the use of anthropometric indicators that were shown to be effective in the present study.

One limitation of this study was inherent to its cross-sectional design. Thus, it was not possible to determine cause-and-effect relation-ships, but only to report associations. Another limitation lay in the limited number of individuals included in this study. This was mainly because of the large number of individuals with diabetes or with abnormal fasting plasma glucose who were being treated at the outpatient medical clinics of Hospital do Servidor Público Estadual de São Paulo, or the high number of non-diabetic individuals who were using oral hypoglycemic agents, under clinical conditions such as non-alcoholic steatohepatitis or metabolic syndrome, which constituted exclusion criteria in the present study.

## CONCLUSION

The most promising anthropometric indicators for assessing IS were BMI, WHtR and WC, and the best cutoff points were 33.3 kg/m^2^, 0.67 and 100 cm, respectively. We also observed an important correlation between WHtR and IR, evaluated through the HOMA-IR index, among overweight or obese females and in non-diabetic females as well, and the best cutoff point was 0.70. These indicators involve simple, fast and easily interpreted anthropometric assessments, which may form an alternative to the HOMA-IS and HOMA-IR indices in clinical practice.

## References

[1] Wild S Roglic G Green A Sicree R King H. Global prevalence of diabetes: estimates for the year 2000 and projections for 2030. Diabetes Care. 2004;27(5):1047-53. 15111519 10.2337/diacare.27.5.1047

[2] Harris MI Klein R Welborn TA Knuiman MW. Onset of NIDDM occurs at least 4-7 yr before clinical diagnosis. Diabetes Care. 1992;15(7):815-9. 1516497 10.2337/diacare.15.7.815

[3] Haffner SM Stern MP Hazuda HP Mitchell BD Patterson JK. Cardiovascular risk factors in confirmed prediabetic individuals. Does the clock for coronary heart disease start ticking before the onset of clinical diabetes? JAMA. 1990;263(21):2893-8. 2338751 10.1001/jama.263.21.2893

[4] UK Prospective Diabetes Study 6. Complications in newly diagnosed type 2 diabetic patients and their association with different clinical and biochemical risk factors. Diabetes Res. 1990;13(1):1-11. 2097090

[5] Glucose tolerance and mortality: comparison of WHO and American Diabetes Association diagnostic criteria. The DECODE study group. European Diabetes Epidemiology Group. Diabetes Epidemiology: Collaborative analysis of Diagnostic criteria in Europe. Lancet. 1999;354(9179):617-21. 10466661

[6] DECODE Study Group, the European Diabetes Epidemiology Group. Glucose tolerance and cardiovascular mortality: comparison of fasting and 2-hour diagnostic criteria. Arch Intern Med. 2001;161(3):397-405. 11176766 10.1001/archinte.161.3.397

[7] Pan XR Li GW Hu YH. Effects of diet and exercise in preventing NIDDM in people with impaired glucose tolerance. The Da Qing IGT and Diabetes Study. Diabetes Care. 1997;20(4):537-44. 9096977 10.2337/diacare.20.4.537

[8] Tuomilehto J Lindström J Eriksson JG. Prevention of type 2 diabetes mellitus by changes in lifestyle among subjects with impaired glucose tolerance. N Engl J Med. 2001;344(18):1343-50. 11333990 10.1056/NEJM200105033441801

[9] Knowler WC Barrett-Connor E Fowler SE. Reduction in the incidence of type 2 diabetes with lifestyle intervention or metformin. N Engl J Med. 2002;346(6):393-403. 11832527 10.1056/NEJMoa012512 PMC1370926

[10] Abdul-Ghani MA Jenkinson CP Richardson DK Tripathy D DeFronzo RA. Insulin secretion and action in subjects with impaired fasting glucose and impaired glucose tolerance: results from the Veterans Administration Genetic Epidemiology Study. Diabetes. 2006;55(5):1430-5. 16644701 10.2337/db05-1200

[11] Faerch K Vaag A Holst JJ. Natural history of insulin sensitivity and insulin secretion in the progression from normal glucose tolerance to impaired fasting glycemia and impaired glucose tolerance: the Inter99 study. Diabetes Care. 2009;32(3):439-44. 19056613 10.2337/dc08-1195 PMC2646025

[12] Chang SA Kim HS Yoon KH. Body mass index is the most important determining factor for the degree of insulin resistance in non-obese type 2 diabetic patients in Korea. Metabolism. 2004;53(2):142-6. 14767863 10.1016/s0026-0495(03)00314-7

[13] Ybarra J Sanchez-Hernandez J Pou JM. Anthropometrical measures are easily obtainable sensitive and specific predictors of insulin resistance in healthy individuals. Prevention and Control. 2005;1(2):175-81. Available from: http://www.journals.elsevierhealth.com/periodicals/precon/article/S1573-2088(05)00034-6/abstract. Accessed in 2010 (Nov 4).

[14] Pitanga FJG Lessa I. Indicadores antropométricos de obesidade como instrumento de triagem para risco coronariano elevado em adultos na cidade de Salvador – Bahia [Anthropometric indexes of obesity as an instrument of screening for high coronary risk in adults in the city of Salvador-Bahia]. Arq Bras Cardiol. 2005;85(1):26-31. 16041451 10.1590/s0066-782x2005001400006

[15] Ho SY Lam TH Janus ED Hong Kong Cardiovascular Risk Factor Prevalence Study Steering Committee. Waist to stature ratio is more strongly associated with cardiovascular risk factors than other simple anthropometric indices. Ann Epidemiol. 2003;13(10):683-91. 14599732 10.1016/s1047-2797(03)00067-x

[16] Valdez R Seidell JC Ahn YI Weiss KM. A new index of abdominal adiposity as an indicator of risk for cardiovascular disease. A cross-population study. Int J Obes Relat Metab Disord. 1993;17(2):77-82. 8384168

[17] Sherwin RS. Diabetes mellitus. In: Goldman L Ausiello D, editors. Cecil Text Book of Medicine. 22 ed. Rio de Janeiro: Elsevier; 2005. p. 1658-92.

[18] Wilmore JH. Body composition in sport and exercise: directions for future research. Med Sci Sports Exerc. 1983;15(1):21-31. 6341750

[19] National Cholesterol Education Program (NCEP) Expert Panel on Detection, Evaluation, and Treatment of High Blood Cholesterol in Adults (Adult Treatment Panel III). Third Report of the National Cholesterol Education Program (NCEP) Expert Panel on Detection, Evaluation, and Treatment of High Blood Cholesterol in Adults (Adult Treatment Panel III) final report. Circulation. 2002;106(25):3143-421. 12485966

[20] Chobanian AV Bakris GL Black HR. Seventh report of the Joint National Committee on Prevention, Detection, Evaluation, and Treatment of High Blood Pressure. Hypertension. 2003;42(6):1206-52. 14656957 10.1161/01.HYP.0000107251.49515.c2

[21] Pouliot MC Després JP Lemieux S. Waist circumference and abdominal sagittal diameter: best simple anthropometric indexes of abdominal visceral adipose tissue accumulation and related cardiovascular risk in men and women. Am J Cardiol. 1994;73(7):460-8. 8141087 10.1016/0002-9149(94)90676-9

[22] Vasques ACJ Rosado LEFPL Alfenas RCG Geloneze B. Análise crítica do uso dos índices do Homeostasis Model Assessment (HOMA) na avaliação da resistência à insulina e capacidade funcional das células-beta pancreáticas: [revisão] [Critical analysis on the use of the homeostasis model assessment (HOMA) indexes in the evaluation of the insulin resistance and the pancreatic beta cells functional capacity: [review]]. Arq Bras Endocrinol Metabol. 2008;52(1):32-9. 18345394 10.1590/s0004-27302008000100006

[23] Geloneze B Vasques ACJ Stabe CFC. Índices HOMA1-IR e HOMA2-IR para identificação de resistência à insulina e síndrome metabólica: Estudo Brasileiro de Síndrome Metabólica (BRAMS) [HOMA1-IR and HOMA2-IR indexes in identifying insulin resistance and metabolic syndrome: Brazilian Metabolic Syndrome Study (BRAMS)]. Arq Bras Endocrinol Metabol. 2009;53(2):281-7. 19466221 10.1590/s0004-27302009000200020

[24] Hanley JA McNeil BJ. A method of comparing the areas under receiver operating characteristic curves derived from the same cases. Radiology. 1983;148(3):839-43. 6878708 10.1148/radiology.148.3.6878708

[25] Rodrigues SL Baldo MP Sá Cunha R. Anthropometric measures of increased central and overall adiposity in association with echocardiographic left ventricular hypertrophy. Hypertens Res. 2010;33(1):83-7. 19911003 10.1038/hr.2009.188

[26] Zhou Z Hu D Chen J. Association between obesity indices and blood pressure or hypertension: which index is the best? Public Health Nutr. 2009;12(8):1061-71. 18778533 10.1017/S1368980008003601

[27] Decoda Study Group Nyamdorj R Qiao Q. BMI compared with central obesity indicators in relation to diabetes and hypertension in Asians. Obesity (Silver Spring). 2008;16(7):1622-35. 18421260 10.1038/oby.2008.73

[28] Nyamdorj R Qiao Q Söderberg S. Comparison of body mass index with waist circumference, waist-to-hip ratio, and waist-to-stature ratio as a predictor or hypertension incidence in Mauritius. J Hypertens. 2008;26(5):866-70. 18398327 10.1097/HJH.0b013e3282f624b7

[29] Vasques ACJ Rosado LEFPL Rosado GP. Habilidade de indicadores antropométricos e de composição corporal em identificar a resistência à insulina [Predictive ability of anthropometric and body composition indicators in the identification of insulin resistance]. Arq Bras Endocrinol Metabol. 2009;53(1):72-9. 19347188 10.1590/s0004-27302009000100011

[30] Ohlson LO Larsson B Svärdsudd K. The influence of body fat distribution on the incidence of diabetes mellitus. 13.5 years of follow-up of the participants in the study of men born in 1913. Diabetes. 1985;34(10):1055-8. 4043554 10.2337/diab.34.10.1055

[31] Prineas RJ Folsom AR Kaye SA. Central adiposity and increased risk of coronary artery disease mortality in older women. Ann Epidemiol. 1993;3(1):35-41. 8287154 10.1016/1047-2797(93)90007-q

[32] Rexrode KM Carey VJ Hennekens CH. Abdominal adiposity and coronary heart disease in women. JAMA. 1998;280(21):1843-8. 9846779 10.1001/jama.280.21.1843

[33] Snijder MB van Dam RM Visser M Seidell JC. What aspects of body fat are particularly hazardous and how do we measure them? Int J Epidemiol. 2006;35(1):83-92. 16339600 10.1093/ije/dyi253

[34] Goodpaster BH Krishnaswami S Resnick H. Association between regional adipose tissue distribution and both type 2 diabetes and impaired glucose tolerance in elderly men and women. Diabetes Care. 2003;26(2):372-9. 12547865 10.2337/diacare.26.2.372

[35] Boyko EJ Fujimoto WY Leonetti DL Newell-Morris L. Visceral adiposity and risk of type 2 diabetes: a prospective study among Japanese Americans. Diabetes Care. 2000;23(4):465-71. 10857936 10.2337/diacare.23.4.465

[36] Lean ME Han TS Morrison CE. Waist circumference as a measure for indicating need for weight management. BMJ. 1995;311(6998):158-61. 7613427 10.1136/bmj.311.6998.158 PMC2550221

[37] Hsieh SD Yoshinaga H. Do people with similar waist circumference share similar health risks irrespective of height? Tohoku J Exp Med. 1999;188(1):55-60. 10494900 10.1620/tjem.188.55

